# Genetic Variation of Morphological Traits and Transpiration in an Apple Core Collection under Well-Watered Conditions: Towards the Identification of Morphotypes with High Water Use Efficiency

**DOI:** 10.1371/journal.pone.0145540

**Published:** 2015-12-30

**Authors:** Gerardo Lopez, Benoît Pallas, Sébastien Martinez, Pierre-Éric Lauri, Jean-Luc Regnard, Charles-Éric Durel, Evelyne Costes

**Affiliations:** 1 Institut National de la Recherche Agronomique, UMR Amélioration Génétique et Adaptation des Plantes méditerranéennes et tropicales, Campus Cirad, Montpellier, France; 2 Montpellier SupAgro, UMR Amélioration Génétique et Adaptation des Plantes méditerranéennes et tropicales, Campus Cirad, Montpellier, France; 3 Institut National de la Recherche Agronomique, UMR Institut de Recherche en Horticulture et Semences, Beaucouzé, France; Wuhan Botanical Garden of Chinese Academy of Sciences, CHINA

## Abstract

Water use efficiency (WUE) is a quantitative measurement which improvement is a major issue in the context of global warming and restrictions in water availability for agriculture. In this study, we aimed at studying the variation and genetic control of WUE and the respective role of its components (plant biomass and transpiration) in a perennial fruit crop. We explored an INRA apple core collection grown in a phenotyping platform to screen one-year-old scions for their accumulated biomass, transpiration and WUE under optimal growing conditions. Plant biomass was decompose into morphological components related to either growth or organ expansion. For each trait, nine mixed models were evaluated to account for the genetic effect and spatial heterogeneity inside the platform. The Best Linear Unbiased Predictors of genetic values were estimated after model selection. Mean broad-sense heritabilities were calculated from variance estimates. Heritability values indicated that biomass (0.76) and WUE (0.73) were under genetic control. This genetic control was lower in plant transpiration with an heritability of 0.54. Across the collection, biomass accounted for 70% of the WUE variability. A Hierarchical Ascendant Classification of the core collection indicated the existence of six groups of genotypes with contrasting morphology and WUE. Differences between morphotypes were interpreted as resulting from differences in the main processes responsible for plant growth: cell division leading to the generation of new organs and cell elongation leading to organ dimension. Although further studies will be necessary on mature trees with more complex architecture and multiple sinks such as fruits, this study is a first step for improving apple plant material for the use of water.

## Introduction

Water use efficiency (WUE) is a quantitative measurement of how much biomass is produced for a given amount of water transpired [1–2]. As feeding the increasing world population with limited water resources progressively becomes a major issue, the improvement of plant WUE has focused the attention of researchers [3–4]. Plant breeding could be an appropriate way to improve WUE since biomass accumulation, transpiration, and WUE have been reported to be under genetic control [5–7]. Moreover, plants may develop different strategies for increasing WUE such as (i) the increase in the leaf photosynthetic capacity [8], (ii) the reduction in leaf conductance [1,8] and plant water potential gradient from the roots to the leaves [9], (iii) the favourable phenology to avoid high evaporative demand [3, 8], (iv) the capability to partition more of the assimilated carbohydrates into the harvest product [8,10], and (v) the development of deeper root system [10].

The genetic diversity and control of WUE have been studied in annual crops such as cereals to identify the best performing genotypes [8,10]. Conversely, the identification of genotypes with high WUE has not yet been engaged for important perennial crops worldwide such as apple (*Malus × domestica* Borkh). In apple, most breeding programs have been dedicated to select resistant genotypes to pests and diseases with high fruit quality [11]. The identification of genotypes with high WUE has only been investigated for a limited number of commercial varieties [12–14]. Only one study has evaluated the genetic variability of WUE in a segregating population of 125 apple genotypes [15]. However, this study focused on intrinsic WUE that was determined from the ratio leaf carbohydrate assimilation to leaf conductance using instantaneous gas exchange measurements. These instantaneous measurements have been extensively used in plant studies because measuring whole-plant WUE and for a long period of time is difficult and tedious [2]. Recently, the evaluation of WUE at more integrated scale and on large populations has been strongly favoured by the development of high-throughput phenotyping technologies (HTPT) [7,16–18].

In this study we aimed at evaluate the amplitude of the genetic variation of WUE and its components (biomass and transpiration) as well as their heritabilities in an apple core collection using HTPT. While information is available on the heritability of physiological traits related with water use such as xylem hydraulic efficiency, stomatal conductance, and leaf carbohydrate assimilation in bi-parental population [15, 19], no study has been designed to determine the heritability for whole-plant biomass, transpiration and WUE. Heritability values for whole-plant WUE are very scant in the literature and only some values have been reported for 148 recombinant inbred lines in sunflower (*Helianthus annuus L*.) [1], and 47 wild barley (*Hordeum vulgare* L.) introgression lines [7]. In our study, we selected a core collection because it enables to enlarge the within-species allelic diversity [20]. The INRA apple core collection was built to generate an optimized representation of old French and European dessert apple germplasms [21]. We used one-year-old plants grown under controlled environmental conditions to facilitate the initial screening for whole-plant WUE, assuming that phenotyping plants in early stages of growth may assist in predicting adult plant performance [17] and because growing the plants under controlled environmental conditions is relevant when studying the genetic control of plant traits [18].

As plant biomass being an important component of WUE and due to the potential variation in organ morphology reported in apple [13, 22–23], we considered also important to identify individual morphological traits correlated with plant biomass and morphotypes within the core collection. Previous studies have been performed to determine architectural groups in apple [23, 24] but the relationships between these architectural groups and their use of water has not been investigated.

Considering the above issues, the specific objectives of this research on the INRA core collection were: i) to determine the genetic variation and heritability of whole-plant biomass, transpiration, WUE, and related descriptors of plant morphology, and ii) to identify different plant morphotypes within the core collection and explore their functionality in terms of use of water.

## Material and Methods

### Plant material

In this study, an INRA apple core collection with 278 genotypes [21] was used. The collection was planted at the INRA experimental unit ‘DiaScope’ in Montpellier, France (43°36ʹ N, 03°58ʹ E) during winter 2014. In February 2014, one-year-old shoots for 193 genotypes were sampled and grafted onto M9 rootstock to generate eight replicates for each genotype. This plant material was complemented with 112 plants obtained from four genotypes with contrasting plant morphology issued from the ‘Starkrimson’ × ‘Granny Smith’ cross [23]. These complementary plants were necessary during the phenotyping phase of the experiment to construct allometric relationships between plant images parameters and measured plant biomass without destroying plants of the core collection. From April 2, all plants were grown in a greenhouse located at the ‘DiasScope’ unit, in nine litre pots filled with a 30:70 (v/v) mixture of a loamy soil and organic compost until a total weight of 4500 g. Plants were irrigated until drainage at the time of planting. After bud break (~ April 12), one shoot per plant was maintained and stakes facilitated their vertical growth. Plants were grown to ensure optimal growth, including automated sub-surface irrigation three times per week, fertilization, weed removal, and pest management until June 3.

### Phenotyping platform

On June 3, all plants were transferred into a greenhouse equipped with HTPT located in Montpellier (PhenoArch). At this time plants had a mean value of 18.9 ± 8.7 leaves. Plants were grown in PhenoArch platform from June 4 until July 19. PhenoArch conveyors allowed the movement of plants within the greenhouse, facilitating the repeated measurements of soil water content (SWC) from pot weighing and plant biomass and leaf area from processed images. A network of eight sensors measured air temperature and humidity (HMP45, Campbell Scientific), and PAR (SKP 215, Campbell Scientific) every minute. Mean values were computed and stored in a database every 15 min. During the experiment, from June 4 until July 19, mean air temperature, humidity, and vapour pressure deficit (VPD) were similar from day to day with respective values of 24.77°C, 64.85%, and 1.13 kPa during the day (S1 Fig). Maximum PAR values was the most variable climatic variables among days and oscillated between 300 and 745 μmol m^−2^ s^−1^ (S1 Fig).

### Experimental design

The 1544 plants from the collection, the 112 complementary plants and 24 pots containing only soil to determine evaporation were organised in 28 lines x 60 rows, with a spacing of 40 and 20 cm, respectively. The complementary plants were located in the extremity of the experimental design (rows one, two, 59 and 60 for all the lines). The other plants and pots were distributed over the rest of the greenhouse according to a matched-pairs experimental design with four block-replicates. For each block, two replicates of the same genotype were located in the same row and within two consecutive lines. Plant biomass and leaf area was monitored for all the plants while plant transpiration and water use efficiency was monitored for four replicates / genotype, i.e. one genotype per block.

### Measurements of plant biomass and leaf area

Biomass and total leaf area for all the plants were estimated every two days using plant images and allometric relationships [16]. Three plant images were taken every two days for each plant by using a 3D Scanalyzer (LemnaTec, Wuüerselen, Germany), one image from the side of each plant at two different rotations angles (0° and 90° side views) and a top view (S2 Fig). For each picture, three parameters were computed using the LemnaGrid software (LemnaTec, Wuüerselen, Germany): Object Sum Area (OSA), which corresponds to the number of all pixels that have been identified as part of the plant; Object Extend (OE), that represents the number of pixels of the width and height of the bounding box that surrounds the plant; and Convex Hull Circumference (CHC), which corresponds to the number of pixels of the area of the smallest convex envelope that contains all pixels that have been identified as part of the plant (S3 Fig).

Multiple linear regressions were used to evaluate the relationship between the image parameters and measured biomass and leaf area as previously used by [16]:

$$\text{Plant biomass}={\;\beta }_{0}+\left(\beta_{1}\times \text{OSA}\_0{}^{\circ}\right)+\left(\beta_{2}\times \text{OSA}\_90{}^{\circ}\right)+\left(\beta_{3}\times \text{OSA}\_\text{top}\right)+\left(\beta_{4}\times \text{OE}\_0{}^{\circ}\right)+\left(\beta_{5}\times \text{OE}\_90{}^{\circ}\right)+\left(\beta_{6}\times \text{OE}\_\text{top}\right)+\left(\beta_{7}\times \text{CHC}\_0{}^{\circ}\right)+\left(\beta_{8}\times \text{CHC}\_90{}^{\circ}\right)+\left(\beta_{9}\times \text{CHC}\_\text{top}\right)$$ (1)

$$\text{Plant leaf area}=\beta_{0}+\left(\beta_{1}\times \text{OSA}\_0{}^{\circ}\right)+\left(\beta_{2}\times \text{OSA}\_90{}^{\circ}\right)+\left(\beta_{3}\times \text{OSA}\_\text{top}\right)$$ (2)

where ß_0_ represents the coefficient for the intercept, and ß_1_ to ß_9_ the regression coefficients for image parameters.

**Fig 1:**
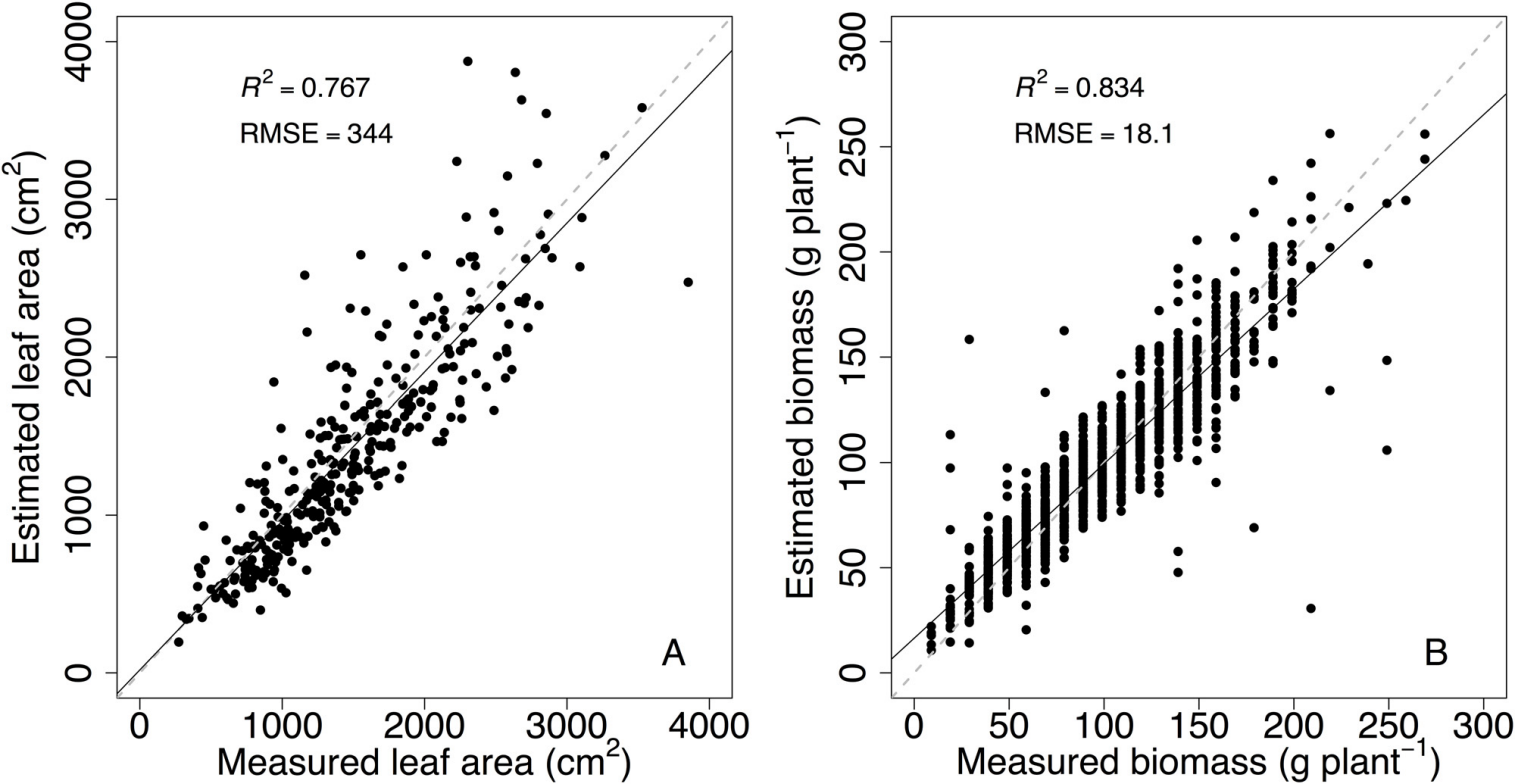
Relationships between measured and estimated whole-plant leaf area (A) and plant biomass (B) at the end of the experiment. Measured plant biomass was obtained after weighing all the plants of the collection. Measured plant leaf area was obtained after measuring the leaf area for all the leaves for two replicates for each genotype. Estimated values were determined using non-destructive phenotyping technologies based on plant imaging. Each point represents one plant. Dashed lines indicate the 1:1 slope and the straight lines represent the regression line between measured and estimated values.

At the end of the experiment (19 July), the regression coefficients of Eqs (1) and (2) were respectively calculated by weighing plant biomass for all the plants and measuring the total leaf area for two replicates of each genotype (LI-COR 3100, Area Meter; Lincoln, NE, USA). The allometric relationships for total plant leaf area and biomass had a root mean square error of 344 cm^2^ and 18 g, respectively (Fig 1).

Accumulated plant biomass and increase in plant leaf area during the experiment (A_Bio and A_LA) were calculated as the difference between values at the beginning (i_Bio and i_LA) and at the end (f_Bio and f_LA) of the experiment (Table 1).

**Table 1:** List of morphological and physiological traits collected from an apple core collection.

Trait	Abv.	Formula	Determination
*Whole-plant morphology*			
Initial biomass (g)	i_Bio		Plant images
Final biomass (g)	f_Bio		Plant images
Accumulated biomass (g)	A_Bio	f_Bio—i_Bio	
Initial leaf area (cm^2^)	i_LA		Plant images
Final leaf area (cm^2^)	f_LA		Plant images
Accumulated leaf area (cm^2^)	A_LA	f_LA—i_LA	
Number of leaves (no.)	Nb_L		Measured
Height (cm)	H		Measured
Basal shoot diameter (mm)	BS_diam		Measured
*Metamer morphology*			
Maximum internode length (mm)	IN_L_max		Measured
Internode diameter (mm)	IN_diam		Measured
Internode dry weight (g)	IN_DW		Measured
Internode volume (mm^3^)	IN_volume	$\pi \text{ x}\left(\frac{\text{IN}\_{\text{diam}}^{2}}{2}\right)\text{x IN}\_\text{L}\_\text{max}$	
Internode density (g mm^-3^)	IN_density	$\frac{\text{IN}\_\text{DW}}{\text{IN}\_\text{volume}}$	
Leaf area (cm^2^)	Leaf_area		Measured
Leaf dry weight (g)	Leaf_DW		Measured
Specific leaf weight (g cm^-2^)	SLW	$\frac{\text{Leaf}\_\text{DW}}{\text{Leaf}\_\text{area}}$	
Leaf:shoot ratio	Leaf:shoot	$\frac{\text{Leaf}\_\text{DW}}{\text{Leaf}\_\text{DW}+\text{IN}\_\text{DW}}$	
*Physiological traits*			
Plant transpiration (g day^-1^)	Plant_T		Pot weight
Leaf transpiration (g cm^-2^ day^-1^)	Leaf_T		Pot weight + plant images
Accumulated transpiration (g)	A_T		Pot weight
Water use efficiency (g g^-1^)	WUE	$\frac{\text{A}\_\text{Bio}}{\text{A}\_\text{T}}$	

### Irrigation management

Irrigation was scheduled for each plant individually to maintain a constant pot target weight by using watering stations with weighing terminals of one g accuracy (ST-Ex, Bizerba, Balingen, Germany) and high-precision irrigation pumps (520U, Watson Marlow, Wilmington, MA, USA). Target pot weight (Wt_target_) was calculated as follows:

$${\text{Wt}}_{\text{target}}={\text{ Wt}}_{\text{rootstock}+\text{scion}}\;+{\text{Wt}}_{\text{pot}}+{\text{Wt}}_{\text{stake}}+{\text{Wt}}_{\text{car}}+\left({\text{Wt}}_{\text{dry soil}}\times \text{SWC}\right)+{\text{Wt}}_{\text{plant}}$$ (3)

where Wt_rootstock+scion_ represents the mean initial weight of the rootstock+scion (67.35 g). This mean value was determined by weighing all the plants for 15 genotypes from the collection before planting. Wt_pot_ (500 g), Wt_stake_ (81 g), and Wt_car_ (1090 g) represent the weight of the pots, stakes and cars where the pots were placed along PhenoArch conveyors, respectively. Wt_dry soil_ that represents the soil dry weight for each pot, was estimated from humidity information of the substrate when pots were filled, which mean value was 1883 g. Soil water content (SWC) for well-watered plants presented in this research was maintained at 1.40 g of water per g of dry soil (70% of field capacity) as previously reported by [16]. Wt_plant_ represents the plant biomass and was the only parameter that varied in Eq (3) during the experiment. To estimate individual Wt_plant_ during the experiment without destroying the plants of the collection, complementary plants allowed the estimation of the regression coefficients of the allometric relationship between plant biomass and pictures parameters presented before. A first relationship was constructed at the beginning of the experiment using 28 plants. The regression coefficients were re-evaluated three times during the experiment to take into account the increase in plant biomass by incorporating 28 plants each time.

### Measurements of plant transpiration and water use efficiency

During the experiment, the PhenoArch platform acquisition system recorded individual pot weight (before and after each irrigation) twice a day. Plant evapotranspiration (ET) between irrigation events was calculated as the difference between the pot weight after a given irrigation and the pot weight before the subsequent irrigation. For each plant, the ET values recorded during two consecutive complete days were summed-up. Soil evaporation (E) for pots with only soil was computed using the same protocol. Plant transpiration (T) was calculated after subtracting the mean values of E of the pots located in the same block from the corresponding ET plant value. Daily T was expressed both as g of water per plant and day (Plant_T) and g of water per cm^2^ of leaf area and day (Leaf_T) dividing the daily transpiration by the imaged-based estimated leaf area. Plant WUE (g g^-1^) was calculated as the ratio of accumulated fresh plant biomass to plant transpiration during the experiment.

### Plant morphology at the end of the experiment

At the end of the experiment, the number of leaves (Nb_L), the plant height (H), and the basal shoot diameter (BS_diam) for each plant was tape-measured manually. For each plant, the metamer with the visually longest internode was sampled, on which, we measured the internode length (IN_L_max), diameter (IN_diam), and dry weight (IN_DW). The area of the leaf of this metamer was measured (Leaf_area) with a leaf area meter (LI-COR 3100, Area Meter; Lincoln, NE, USA). Its dry weight (Leaf_DW) as well as IN_DW were obtained after drying the samples in a forced-air draft oven at 65°C. We also calculated the internode volume assuming a cylindrical shape for the internode (IN_volume), the internode density (mass of dry matter per unit internode volume, IN_density), the specific leaf weight (mass of dry matter per unit leaf area, SLW), and the ratio between the leaf and the metamer weight (leaf:shoot ratio) (Table 1).

### Analysis of data and statistical analysis

All the analyses were performed using the R software [25]. Analyses of data were performed for 183 genotypes because most of the plants for ten genotypes died during the experiment and were thus not considered in final analyses.

For all the traits evaluated in this study (Table 1), nine mixed effect models were developed to account for the genetic effect, considered as random, and the spatial heterogeneity inside the platform (S1 Table). The different models differed only in the way spatial effects were took into account. The modelling of spatial effects was performed using the ‘asreml’ package of R software [26]. Model 1, did not take into account any spatial effects, whereas models two, three and four took into account a fixed effect of blocks, lines, and rows, respectively. Models five, six, and seven considered a two-dimensional structures for the residuals using a first order autoregressive correlation models on the line direction only (model 5), on the row direction only (model 6) or in both directions (model 7). Two metric based models were also tested: a two-dimensional exponential model (model 8) and a two dimensional Gaussian model (model 9) to model the correlation between residuals. These models were based on kriging methods and assumed a decrease in the spatial correlation between residuals with increasing distance [27].

The Best Linear Unbiased Predictors (BLUPs) of genetic values were estimated after having selected the model that minimized the Bayesian Information Criterion (BIC). For each genotype the genetic value was calculated as the sum of the population mean and its corresponding BLUPs.

Variance estimates of the selected models were used to compute the mean broad-sense heritability (H^2^) according to [16]:

$${\text{H}}^{2}=\;\frac{\sigma_{\text{G}}^{2}}{\left[\sigma_{\text{G}}^{2}+\left(\frac{\sigma_{\text{R}}^{2}}{\text{n}}\right)\right]}$$ (4)

where σ^2^_G_ is the genetic variance, σ^2^_R_ the residual variance, and n the number of replicates per genotype.

The genetic correlation between traits was evaluated using the bootstrap resampling method provided by the ‘boot’ package of R software. Using this method, the Pearson coefficient of correlation (r) and the corresponding p-value at a probability of 5% were estimated as the mean values of one thousand random samples of the population. This methodology limits the influence of the outlier’s genotypes on the correlation [28].

To identify plant morphotypes, the genetic values for all the morphological traits used for the description of the whole plant (f_Bio, f_LA, H, Nb_L, and BS_diam) and selected traits for the metamer with the longest internode were used to identify clusters by using a Hierarchical Ascendant Classification (HAC) [29] with the HAC package of R software. Descriptors of the metamer morphology that presented high correlation with other traits were discarded for the HAC to avoid redundancy on cluster definition as suggested by [23]. Variables with low H^2^ (H^2^ < 0.5) were also discarded to minimize the use of descriptors with a low genetic control. To determine how the morphological traits discriminated the clusters in the HAC, a one-way analysis of variance (ANOVA) with a cluster effect was performed for each trait. Tukey’s test was then applied to separate least square means that differed significantly. Statistical significance was established for *P*<0.05. Additionally, mean values for Plant_T, Leaf_T, and WUE that were not used for the HAC were calculated for the clusters. On these physiological traits, ANOVA and Tukey’s tests were also performed to determine statistical differences between clusters.

## Results

### Spatial heterogeneity in PhenoArch, heritability and genetic coefficient of variations

**Fig 2:**
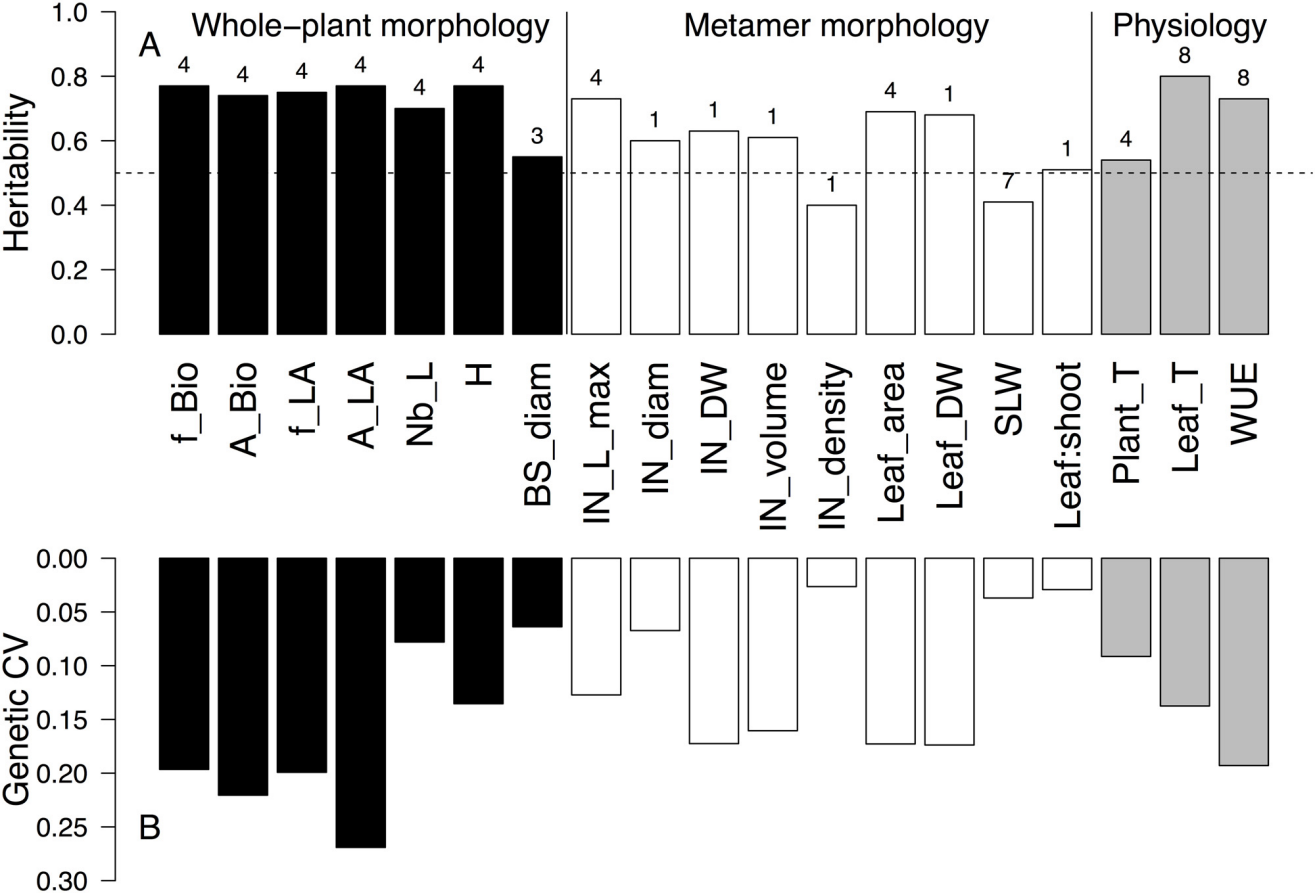
Broad-sense heritability (A) and genetic coefficient of variation (CV) (B) for the genetic values of morphological and physiological traits for an apple core collection. For each trait, the number of the model selected according to the Bayesian Information Criterion (BIC) minimization is presented at the top of heritability bars. Refer to Table 1 for traits abbreviation and to S1 Table for model details.

Among the nine mixed models that were tested to take into account the spatial heterogeneity in PhenoArch and to estimate the genetic values, only five models were selected by BIC minimization (S1 Table). The model containing the fixed effect of row position (model 4) was the most often selected (Fig 2A and S1 Table). Model 4 was selected for nine traits (IN_L_max, Leaf_area, Plant_T, and all the morphological traits at the whole-plant level, except BS_diam). The model that did not include any spatial effect (model 1) was selected for six descriptors of the metamer morphology (IN_diam, IN_DW, IN_volume, IN_density, Leaf_DW, and Leaf:shoot ratio) (Fig 2A). One metric based model (Model 8) was selected for Leaf_T and WUE (Fig 2A). Models containing an autoregressive model for line position (model 3) and row and line position (model 7) were selected only for BS_diam and SLA, respectively (Fig 2A). Although the model selection results indicates that some traits exhibited a certain variability depending on the position of the plants in the platform, BIC and H^2^ values for the selected models and model 1 were close (S1 Table), indicating that the environmental variability within PhenoArch was low.

All the traits at the whole plant level had H^2^ values between 0.7 and 0.8, except BS_diam that had a H^2^ of 0.55 (Fig 2A). At the metamer level H^2^ values were generally lower (between 0.5 and 0.73) and two traits had H^2^ values lower than 0.5: IN_density and SLW (Fig 2A). For the physiological traits, Leaf_T and WUE reached high H^2^ values (0.80 and 0.73 respectively) whereas Plant_T had a H^2^ value of 0.54 (Fig 2A). The traits with the highest genetic coefficient of variation belonged to the whole plant morphology variables with two variables displaying coefficient of variation higher than 0.20 (A_Bio = 0.22 and A_LA = 0.27) (Fig 2B). Traits of the metamer morphology reached lower levels of variation with some of them (IN_diameter, IN_density, SLA, and leaf:shoot ratio) displaying coefficients of variation lower than 0.10 (Fig 2B). For the physiological traits, Plant_T, Leaf_T and WUE had genetic coefficient of variation of respectively 0.09, 0.14, and 0.19 (Fig 2B).

### Genetic correlation between morphological traits at the whole-plant level

Initial and final values of plant biomass and leaf area were highly correlated. The coefficient of correlations (r) between A_Bio and f_Bio and A_LA and f_LA were equal to 0.95 and 0.92, respectively (Table 2). The final plant biomass and leaf area during the experiment were correlated with their increase during the experiment (r = 0.95 between f_Bio and A_Bio and r = 0.92 between A_LA and f_LA). The correlation analysis also revealed significant correlations but of lower intensity between the initial values of biomass and leaf area and their increase during the experiment (r = 0.64 between A_Bio and i_Bio and r = 0.53 between A_LA and i_LA). High r values were observed between f_Bio and H (0.85) and f_Bio and f_LA (0.76) (Table 2). Other correlations had r values lower than 0.60, indicating the existence of certain variability within the collection (Table 2). The weakest correlations were observed between BS_diam and H (r = 0.26) and BS_diam and Nb_L (r = 0.31) (Table 2).

**Table 2:** Genetic correlations between morphological traits at the whole-plant level for an apple core collection.

	Initial values		Accumulated values		Final values			
	i_Bio	i_LA	A_Bio	A_LA	f_Bio	f_LA	Nb_L	H
i_Bio								
i_LA	0.79***							
A_Bio	0.64***	0.43***						
A_LA	0.52***	0.53***	0.81***					
f_Bio	0.85***	0.62***	0.95***	0.70***				
f_LA	0.71***	0.81***	0.69***	0.92***	0.76***			
Nb_L	0.40***	0.38***	0.42***	0.44***	0.45***	0.47***		
H	0.70***	0.40***	0.82***	0.52***	0.85***	0.54***	0.54***	
BS_diam	0.60***	0.68***	0.37**	0.40***	0.46***	0.57***	0.31**	0.26*

The significance of each Pearson’s r coefficient of correlation is indicated as follows:

* = P<0.05;

** = P<0.01;

*** = P<0.001;

n.s. = not significant. Refer to Table 1 for traits abbreviation.

### Genetic correlation between morphological traits at the metamer level

Internode maximum length, diameter and density were weakly correlated or not correlated among them but they were highly correlated with the internode dry weight (Table 3). Individual leaf area was highly correlated with its dry weight but not with its SLW (Table 3). Although Leaf:shoot ratio was negatively correlated with the internode maximum length, dry weight, and volume and positively correlated with leaf area and dry weight the r values remained low (absolute values between 0.28 and 0.42, Table 3), indicating a combined impact of leaf and internode variables on the value of leaf:shoot ratio. The positive correlations between internode and leaf traits indicated that the dimension of both organs were positively linked but in many cases the r values were lower than 0.45, indicating also the existence of a high variability within the collection (Table 3). Since IN_DW, IN_volume, and Leaf_DW were highly correlated and also correlated with other traits, they were discarded in further analyses to avoid redundancy of variables.

**Table 3:** Genetic correlations between morphological traits at the metamer level for an apple core collection.

	Internode					Leaf		
	IN_L_max	IN_diam	IN_DW	IN_volume	IN_density	Leaf_area	Leaf_DW	SLW
IN_L_max								
IN_diam	n.s.							
IN_DW	0.61***	0.69***						
IN_volume	0.61***	0.76***	0.95***					
IN_density	n.s.	-0.23*	n.s.	n.s.				
Leaf_area	0.31**	0.45**	0.57***	0.55***	n.s.			
Leaf_DW	0.35***	0.51***	0.63***	0.62***	n.s.	0.92***		
SLW	n.s.	0.22*	0.23*	0.25*	n.s.	n.s.	0.27*	
Leaf:shoot	-0.28*	n.s.	-0.41***	-0.36**	n.s.	0.36***	0.37***	n.s.

The significance of each Pearson’s r coefficient of correlation is indicated as follows:

* = P<0.05;

** = P<0.01;

*** = P<0.001;

n.s. = not significant. Refer to Table 1 for traits abbreviation.

### Genetic correlation between morphological traits at the whole-plant and metamer level

There were many positive correlations between traits at the whole-plant and at the metamer level (Table 4). The highest r value were observed between BS_diam and IN_diam (r = 0.76) and plant H and IN_L_max (r = 0.57) (Table 4). f_Bio was significantly correlated with all the descriptors of the metamer morphology, except with SLW (Table 4). The following decreasing ranking for high positive correlations with f_Bio was observed: IN_L_max (r = 0.55), Leaf_area (r = 0.54), and IN_diam (r = 0.44). f_Bio was, however, negatively correlated with the Leaf:shoot ratio (r = -0.24) (Table 4). f_LA was less correlated with the metamer morphology than f_Bio. The f_LA was not correlated with IN_L_max, IN_density, SLW, and Leaf:shoot ratio (Table 4). It was only correlated with Leaf_area (r = 0.58) and IN_diam (r = 0.57). The Nb_L and BS_diam were weakly or not correlated with the descriptors of the internode morphology, except for the high correlation between BS_diam and IN_diam (Table 4).

**Table 4:** Genetic correlations between morphological traits at the whole-plant level and at the metamer level for an apple core collection.

	*Whole-plant*						
	A_Bio	A_LA	f_Bio	f_LA	Nb_L	H	BS_diam
*Metamer*							
IN_L_max	0.54***	n.s.	0.55***	n.s.	n.s.	0.57***	n.s.
IN_D	0.33**	0.48***	0.44**	0.57***	0.28**	n.s.	0.76***
IN_density	0.25*	n.s.	0.21*	n.s.	n.s	0.26*	n.s.
Leaf_area	0.57***	0.59***	0.54***	0.58***	n.s	0.28**	0.39***
SLW	n.s.	n.s.	n.s.	n.s.	n.s	n.s	n.s.
Leaf:shoot	n.s.	n.s.	-0.24*	n.s.	-0.26**	-0.35***	n.s.

The significance of each Pearson’s r coefficient of correlation is indicated as follows:

* = P<0.05;

** = P<0.01;

*** = P<0.001;

n.s. = not significant. Refer to Table 1 for traits abbreviation.

### Genetic correlations between morphological and physiological traits

**Fig 3:**
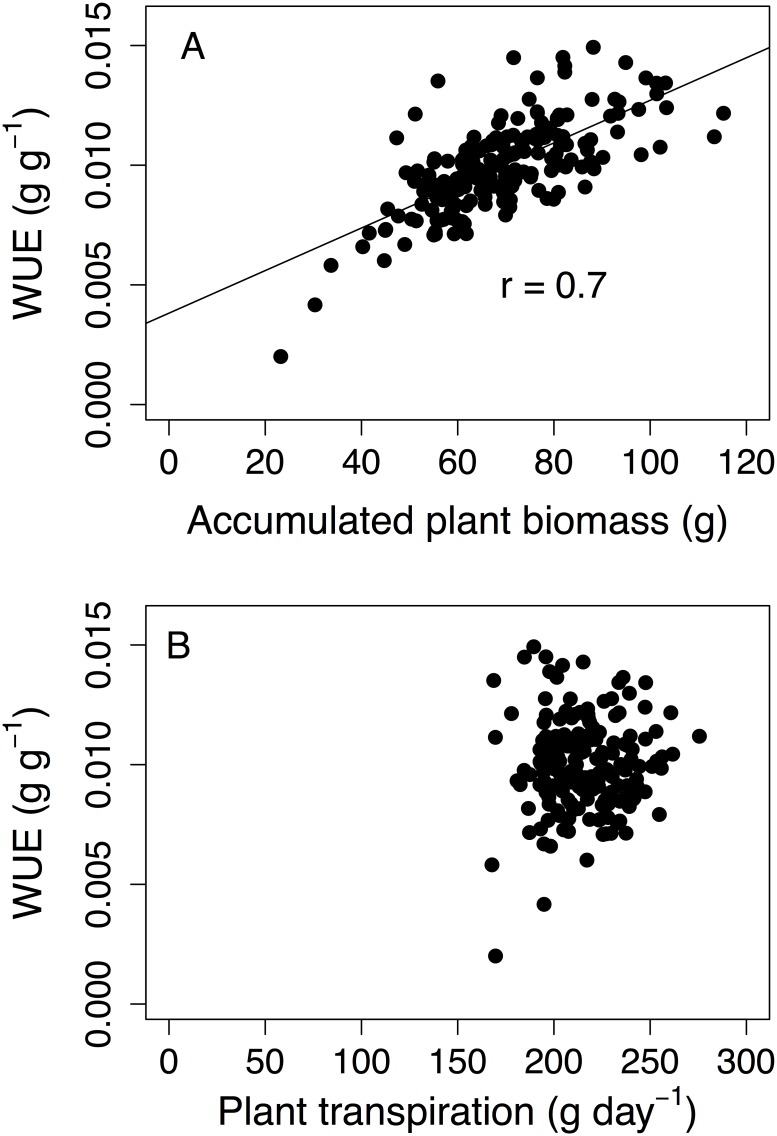
Genetic correlations between accumulated plant biomass and WUE (A), and mean plant transpiration and WUE (B) for an apple core collection. The correlations between traits were evaluated using the bootstrap resampling method. Regression lines were presented for significant correlations. Each point represents the genetic value for one genotype.

Plant_T, Leaf_T, and WUE were not correlated (Table 5). Plant_T was positively correlated with all the descriptors of the whole-plant morphology and three morphological descriptors at the metamer level (IN_L_max, IN_diam, and Leaf_area) (Table 5). The highest r was observed between Plant_T and f_LA (0.81). Leaf_T was negatively correlated with A_LA, f_Bio, f_LA, and BS_diam but the r values were in general lower than those observed between Plant_T and morphological traits (Table 5). The highest absolute r value was observed between Leaf_T and f_LA (-0.54). Leaf_T was not correlated with any descriptor of the metamer morphology (Table 5). Although WUE was calculated as the ratio between accumulated biomass and accumulated transpiration during the experiment, it was only correlated to A_Bio (r = 0.7) (Table 5 and Fig 3). After A_Bio, the following decreasing ranking for positive correlations with WUE was: H (r = 0.55), f_Bio (r = 0.54), IN_L_max (r = 0.45), A_LA (r = 0.35). Two other traits were weakly and negatively correlated with WUE: SLW (r = -0.31), and BS_diam (r = -0.23) (Table 5).

**Table 5:** Genetic correlations between morphological and physiological traits for an apple core collection.

	Physiological traits		
	Plant_T	Leaf_T	WUE
*Whole-plant morphology*			
A_Bio	0.66***	n.s.	0.70***
A_LA	0.70***	-0.39***	0.35**
f_Bio	0.77***	-0.20*	0.54***
f_LA	0.81***	-0.54***	n.s.
Nb_L	0.46***	n.s.	n.s.
H	0.56***	n.s.	0.55***
BS_diam	0.68***	-0.25*	-0.23*
*Metamer morphology*			
IN_L_max	0.26**	n.s.	0.45***
IN_diam	0.68***	n.s.	n.s.
IN_density	n.s.	n.s.	n.s.
Leaf_area	0.62***	n.s.	n.s.
SLW	n.s.	n.s.	-0.31**
Leaf:shoot	n.s.	n.s.	n.s.
*Physiological traits*			
Plant_T			
Leaf_T	n.s.		
WUE	n.s	n.s.	

The significance of each Pearson’s r coefficient of correlation is indicated as follows:

* = P<0.05;

** = P<0.01;

*** = P<0.001;

n.s. = not significant. Refer to Table 1 for traits abbreviation.

**Fig 4:**
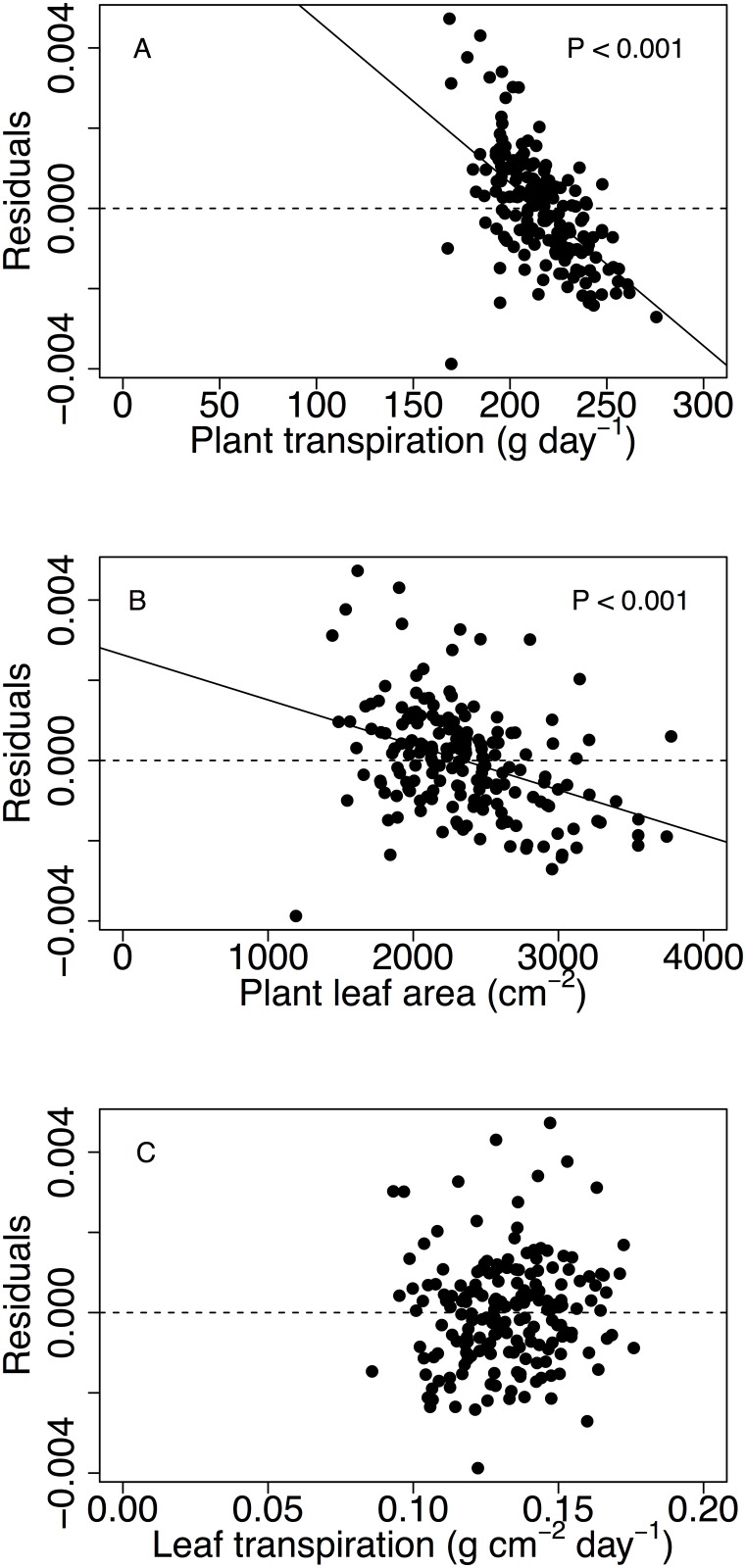
Relationships between plant transpiration (A), plant leaf area at the end of the experiment (B) and leaf transpiration (C) with the Y-axis representing residuals of the relationship between accumulated plant biomass and WUE in Fig 3A. Regression lines were presented for significant correlations. Each point represents the genetic value for one genotype.

Since a very strong relationship between WUE and A_Bio was observed (Fig 3A), correlations between plant transpiration, leaf area, and leaf transpiration and the Y-axis residuals of the linear relationship between WUE (Y-axis variable) and plant biomass (X-axis variable) were investigated. This revealed that the variability in WUE that was not explained by A_Bio was related with Plant_T (Fig 4A) due to the morphological variability in leaf area (Fig 4B) rather than to the variability in the rate of leaf transpiration (Fig 4C).

### Identification of plant morphotypes at the end of the experiment

To identify different plant morphotypes within the core collection, a HAC was performed using five descriptors of the whole-plant morphology (f_Bio, f_LA, Nb_L, H, and BS_diam) and four descriptors for the metamer (IN_L_max, IN_diam, Leaf_area, and Leaf:shoot). The HAC was first partitioned in two main groups (A and B) (S2 Table) which mainly differed by their total leaf area. Group A had higher values than cluster B for all the traits except for the number of leaves (Nb_L) and leaf:shoot ratio (S2 Table). Then the collection was progressively partitioned (S3 Table) until three clusters were distinguished within clusters A and B (Table 6 and S4 Fig). Clusters A1.1, A1.2, and A2 had respectively 17, 20 and 32 genotypes. Cluster A1.1 had high values for all the morphological traits except for the intermediate values of diameters (BS_diam, IN_diam), and leaf:shoot (Table 6). Cluster A2, had lower biomass than cluster A1.1. This was explained by an increase in the number of leaves with shorter internode length (more compact morphotype). Cluster A2 had also high values of BS_diam and IN_diam (Table 6). Cluster A1.2 had lower biomass than cluster A1.1 and A2, which was mainly explained by a reduction in the total number of leaves and total plant length (Table 6). Group B was divided in three clusters: B1.1, B1.2, and B2 with respectively 17, 56 and 41 genotypes (Table 6). These three clusters had smaller individual leaf area than group A. This reduction in individual leaf area produced lower plant biomass in genotypes included in cluster B2 because they also had low values for plant length, total number of leaves, and internode length. Cluster B1.1 and B1.2 were very similar for all the traits (except for basal diameter and internode length) and exhibited intermediate plant biomass due to higher values of plant length, number of leaves, and internode length than those observed in cluster B2.

**Table 6:** Hierarchical ascendant classification (HAC) of an apple core collection in six clusters according to nine morphological traits measured at the end of the experiment. Information of the physiological traits for each group is also presented.

	Clusters					
Traits	A1.1	A1.2	A2	B1.1	B1.2	B2
No. of genotypes	17	20	32	17	56	41
*Morphological traits (whole-plant)*						
f_Bio (g)	147 a	110 c	124 b	113 bc	107 c	85 d
f_LA (cm^2^)	2906 a	2354 b	2878 a	2166 bc	2226 b	1933 c
H (cm)	144 a	119 c	127 bc	130 b	124 bc	102 d
Nb_L (#)	43.98 ab	41.25 c	46.26 a	43.89 b	45.28 ab	40.82 c
BS_diam (mm)	8.67 b	8.49 bc	9.15 a	8.63 b	8.21 cd	8.09 d
*Morphological traits (metamer with the longest internode)*						
IN_L_max (mm)	45.05 a	40.73 b	35.59 cd	41.37 b	35.76 c	33.84 d
IN_diam (mm)	6.87 b	6.67 bcd	7.20 a	6.75 bc	6.51 cd	6.39 d
Leaf_area (cm^-2^)	77.36 a	75.92 ab	70.34 b	57.57 c	59.24 c	57.63 c
Leaf:shoot (g g^-1^)	0.76 b	0.78 a	0.76 b	0.74 c	0.77 b	0.78 a
*Physiological traits*						
Plant_T (g day^-1^)	239 a	218 b	236 a	214 b	211 b	195 c
Leaf _T (g cm^-2^ day^-1^)	0.1276 ab	0.1326 ab	0.1236 ab	0.1387 a	0.1351 a	0.1348 ab
WUE (g g^-1^ day^-1^)	0.0121 a	0.0102 bc	0.0092 c	0.0101 bc	0.0104 b	0.0089 c
*Percentage of reduction relative to cluster A1*.*1*						
f_Bio		-25	-16	-23	-27	-42
Plant_T		-9	-1	-10	-12	-18

For each group and trait, a one-way ANOVA was performed to estimate cluster effects. For each variable, mean values with different letters indicates significant differences between groups according to Tukey’s test and a *P*<0.05. See Table 1 for the interpretation of traits abbreviation.

### Identification of differences in water use between morphotypes

When the mean values for WUE, Plant_T, and Leaf_T, were calculated for each cluster, significant differences in WUE and Plant_T were observed among morphotypes (Table 6). The cluster A1.1 had the maximum values of WUE and Plant_T. Cluster A2 had low values of WUE because there was a lower production of plant biomass for an equivalent Plant_T in comparison with cluster A1.1. Clusters A1.2, B1.1, and B1.2 had similar intermediate values of Plant_T and plant biomass and consequently these three clusters had similar intermediate values of WUE (Table 6). Cluster B2 had the lowest biomass production (reduction of 42% in comparison with cluster A1.1) and Plant_T (reduction of 18% in comparison with cluster A1.1) and consequently its value of WUE was very low (Table 6).

## Discussion

High-throughput phenotyping technologies (HTPT) were used to study the genetic control and variation of whole-plant biomass, leaf area, transpiration and WUE for an INRA apple core collection under well-watered conditions. HTPT allowed the determination of whole-plant transpiration by pot weighing and plant biomass and leaf area from analysis of images (S2 and S3 Figs) with acceptable accuracy (Fig 1). A detailed description of plant morphology was also manually performed to decompose plant biomass into morphological components related to either growth or organ expansion and distinguish morphotypes with contrasted morphology and water use efficiency.

The first objective of this work was to report values of heritability (H^2^) and genetic variation of some morphological and physiological traits at organ and plant scale for the INRA core collection (Fig 2). In our study, all traits had H^2^ values higher than 0.5 and display large genetic variation, except the internode density and the specific leaf weight (Fig 2A). The H^2^ values for traits such as WUE and plant biomass were higher than those observed in other studies in sunflower [1] and rice (*Oryza sativa* L.) [30] that used the same formula to compute H^2^. This could indicate that WUE in apple has a strong genetic control. H^2^ values of morphological traits such as H, IN_L_max, and BS_diam, and Nb_L were also higher than those previously observed in biparental populations in apple [23, 31–33] and olive (*Olea Europea* L.) [34]. These differences in H^2^ could be partly related to larger allelic diversity included in the core collection compared to biparental populations. Other possible explanations for the high H^2^ values in our study are also the low temporal and spatial environmental variability in PhenoArch (S1 Fig and S1 Table) together with the use of many mixed effect models limiting the errors in the estimation of genetic values due to spatial variability (S1 Table). Moreover, four replicates per genotype were used in this study while in other studies less replicates were used [1, 29]. Heritability values also indicated that biomass (0.76) had a higher genetic control than plant transpiration (0.54). The genetic variation of WUE was mainly explained by accumulated biomass and not by plant transpiration (Fig 3 and Table 5) as previously reported for genotypes in sunflower [1], barley [7], and maritime pine [35]. However a lack of relationship between biomass and WUE could be also observed [8], suggesting that for a given amount of water transpired, the acquired carbon may be allocated to different plant organs or processes. The positive correlation between WUE and biomass accumulation in our study indicated how important was to decompose the biomass into morphological components and to have a better understanding of their genetic diversity within the core collection.

In this study the genetic diversity of the core collections was analysed by means of a hierarchical ascendant classification, which led us to identify six groups of genotypes with contrasted morphology (Table 6). Taking into account that differences in leaf photosynthesis rate between apple genotypes are small [36], we hypothesize that differences between morphotypes (Table 6 and S4 Fig) may result from differences in the main processes responsible for plant growth: cell division leading to the generation of new organs (leaves and internodes) and tissues (e.g. wood), and cell elongation leading to organ dimension. These two processes have been shown to have antagonistic effects on the organ final size [37] and are usually considered under the influence of resource allocation to developing organs [38, 39]. It must be noticed that in our study, the difference in biomass between cluster A1.1 and A2 could be due to larger resource allocation to root compartment since these trees were similar with respect to their leaf area but those in A2 group had a lower aerial biomass (Table 6). Collecting detailed physiological data related with plant carbohydrate assimilation and distribution among plant organs for selected genotypes of the core collection with contrasting architecture will constitute a further step to better understand the physiological mechanism that may explain the observed variations.

Another output of this study is that we were able to identify individual morphological traits correlated with WUE. This topic is relevant because the quantitative evaluation of WUE is complex and time-consuming [2]. WUE had a strong positive relationship with two easy-to-measure traits such as plant height and internode maximum length (Table 5). Since these traits are highly heritable (Fig 2A) they could be easily implemented as secondary traits in apple-breeding programs. The internode maximum length has been recently proposed as an important morphological trait due to its stability among different environments [40]. We also identified a group of 17 genotypes with high WUE (Table 6). A remaining issue is the evaluation of the core collection under sub-optimal growing conditions to determine if those genotypes with high WUE under well-watered conditions are also the best performing genotypes under water stress. In future research we also expect to identify genetic polymorphisms associated with WUE. Although understanding the genetic control and variation of WUE in mature plants with more complex architecture and multiple sinks organs such as fruits will also be necessary, this study is a first step for improving apple plant material for the use of water.

## Supporting Information

S1 Table Bayesian Information criterion (BIC) and heritability (H^2^) (in brackets) for the model with the lowest BIC and for model 1 that did not take into account any special effect. When values are only presented for model 1 that means that model 1 had the lowest BIC. In all the models, the genetic effect was considered as a random effect. Refer to Table 1 for traits abbreviation.
(PNG)

S2 Table Hierarchical ascendant classification (HAC) of an apple core collection in two clusters according to morphological traits measured at the end of the experiment. For each group and trait, a one-way ANOVA was performed to estimate the cluster effects. For each variable, mean values with different letters indicates significant differences between groups according to Tukey’s test and a *P*<0.05. Refer to Table 1 for traits abbreviation.
(PNG)

S3 Table Hierarchical ascendant classification (HAC) of an apple core collection in four clusters according to morphological traits measured at the end of the experiment. For each group and trait, a one-way ANOVA was performed to estimate the cluster effects. For each variable, mean values with different letters indicates significant differences between groups according to Tukey’s test and a *P*<0.05. Refer to Table 1 for traits abbreviation.
(PNG)

S1 Fig Patterns of maximum, mean and minimum daily air temperature, photosynthetic active radiation (PAR), air relative humidity, and vapour pressure deficit (VPD) during the experiment in the phenotyping platform. (PDF)

S2 Fig Example of the 0° (A), 90° (B) and top view (C) images collected by the phenotyping platform for one plant of the apple core collection at the end of the experiment. (PDF)

S3 Fig Example of the 0° view of a 3D plant image and parameters derived from the analysis of pictures. A: original plant image; B: ‘Object Sum Area’ (number of all pixels that have been identified as part of the plant); C: Object Extend (number of pixels of the width and height of the bounding box that surrounds the plant); and D: ‘Convex Hull Circumference’ (number of pixels of the area of the smallest convex envelope that contains all pixels that have been identified as part of the plant).
(PDF)

S4 Fig Tree dendogram for a hierarchical ascendant classification of an apple core collection in six clusters according to five traits at the whole-plant level (leaf number, height, biomass, leaf area, and basal diameter) and six traits for the metamer with the longest internode (internode length, diameter and density, leaf area, specific leaf area, and leaf:shoot ratio). For each cluster, an example of a representative plant is presented.
(PDF)

## References

[1] Adiredjo AL, Navaud O, Muños S, Langlade NB, Lamaze T, Grieu P. Genetic control of water use efficiency and leaf carbon isotope discrimination in sunflower (*Helianthus annuus* L.) subjected to two drought scenarios. PLoS ONE 2014; 9: e101218 10.1371/journal.pone.0101218 24992022 PMC4081578

[2] Tardieu F. Plant response to environmental conditions: assessing potential production, water demand, and negative effects of water deficit. Front Physiol. 2013; 4: 17 10.3389/fphys.2013.00017 23423357 PMC3574982

[3] Hamdy A, Ragab R, Scarascia-Mugnozza E. Coping with water scarcity: water saving and increasing water productivity. Irrig. and Drain. 2003; 52: 3–20.

[4] Tester M, Langridge P. Breeding technologies to increase crop production in a changing world. Science 2010; 327: 818–822. 10.1126/science.1183700 20150489

[5] Masle J, Gilmore SR, Farquhar GD. The ERECTA gene regulates plant transpiration efficiency in Arabidopsis. Nature 2005; 436: 866–870. 16007076 10.1038/nature03835

[6] Johnson K, Lenhard M. Genetic control of plant organ growth. New Phytol. 2011; 191: 319–333. 10.1111/j.1469-8137.2011.03737.x 21517873

[7] Neilson EH, Edwards AM, Blomstedt CK, Berger B, Møller BL, Gleadow RM. Utilization of a high-throughput shoot imaging system to examine the dynamic phenotypic responses of a C4 cereal crop plant to nitrogen and water deficiency over time. J. Exp. Bot. 2015; 10.1093/jxb/eru526 PMC4378625 25697789

[8] Condon AG, Richards RA, Rebetzke GJ, Farquhar GD. Breeding for high water-use efficiency. J. Exp. Bot. 2004; 55: 2447–2460. 15475373 10.1093/jxb/erh277

[9] Glenn DM, Scorza R, Bassett C. Physiological and morphological traits associated with increased water use efficiency in the willow-leaf peach. HortScience 2000; 35: 1241–1243.

[10] Slafer GA, Araus MP, Royo C, Del Moral LFG. Promising eco-physiological traits for genetic improvement of cereal yields in Mediterranean environments. Ann. Appl. Biol. 2005; 146: 61–70.

[11] Brown SK, Maloney KE. Genetic improvement of apple: breeding, markers, mapping and biotechnology In: Ferree DC, Warrington IJ, editors. Apples: Botany, production and uses. CAB International; 2003 pp. 31–59.

[12] Massonnet C, Costes E, Rambal S, Dreyer E, Regnard JL. Stomatal regulation of photosynthesis in apple leaves: evidence for different water-use strategies between two cultivars. Ann. Bot. 2007; 100: 1347–1356. 17901058 10.1093/aob/mcm222 PMC2759240

[13] Liu BH, Cheng L, Ma FW, Zou YJ, Liang D. Growth, biomass allocation, and water use efficiency of 31 apple cultivars grown under two water regimes. Agrofor. Syst. 2012; 84: 117–129.

[14] Sun XP, Yan HL, Kang XY, Ma FW. Growth, gas exchange, and water-use efficiency response of two young apple cultivars to drought stress in two scion-one rootstock grafting system. Photosynthetica 2013; 51: 404–410.

[15] Regnard JL, Ducrey M, Porteix E, Segura V, Costes E. Phenotyping apple progeny for ecophysiological traits: how and what for? Acta Hort. 2008; 772: 151–158.

[16] Coupel-Ledru A, Lebon E, Christophe A, Doligez A, Cabrera-Bosquet L, Péchier P, et al Genetic variation in a grapevine progeny (*Vitis vinifera* L. cvs Grenache×Syrah) reveals inconsistencies between maintenance of daytime leaf water potential and response of transpiration rate under drought. J. Exp. Bot. 2014; 65: 6205–6218. 10.1093/jxb/eru228 25381432 PMC4223985

[17] Honsdorf N, March TJ, Berger B, Tester M, Pillen K. High-throughput phenotyping to detect drought tolerance QTL in wild barley introgression lines. PLoS ONE 2014; 9: e97047 10.1371/journal.pone.0097047 24823485 PMC4019662

[18] Yang W, Guo Z, Huang C, Duan L, Chen G, Jiang N, et al Combining high-throughput phenotyping and genome-wide association studies to reveal natural genetic variation in rice. Nature Comm. 2014; 5: 5087. 10.1038/ncomms6087 PMC4214417 25295980

[19] Lauri PE, Gorza O, Cochard H, Martinez S, Celton JM, Ripetti V, et al Genetic determinism of anatomical and hydraulic traits within an apple progeny. Plant Cell Environ. 2011; 34: 1276–1290. 10.1111/j.1365-3040.2011.02328.x 21477120

[20] Brown AHD. Core collections: a practical approach to genetic resources management. Genome 1989; 31: 818–824.

[21] Lassois L, Denancé C, Ravon E, Guyader A, Guisnel R, Hibrand-Saint-Oyant L, et al Genetic diversity, population structure, parentage analysis and construction of core collections in the French apple germplasm based on SSR markers. Plant Mol. Biol. Rep. 2015 10.1007/s11105-015-0966-7

[22] Bassett CL, Glenn DM, Forsline PL, Wisniewski ME, Farrel RE. Characterizing water use efficiency and water deficit responses in apple (*Malus xdomestica* Borkh. and *Malus sieversii* Ledeb.) M. Roem. HortScience 2011; 46: 1909–1084.

[23] Segura V, Cilas C, Laurens F, Costes E. Phenotyping progenies for complex architectural traits: a strategy for 1-year old apple trees (*Malus x domestica* Borkh.). Tree Genet. Genomes 2006; 2: 140–151.

[24] Lespinasse Y. Breeding apple tree: aims and methods In: Rousselle-Bourgeois F, Rousselle P, editors. Proceedings of the joint conference of the EAPR breeding and varietal assessment section and the EUCARPIA potato section; 1992 pp. 103–110.

[25] R Development Core Team. R: a language and environment for statistical computing. R Foundation for Statistical Computing, Vienna, Austria; 2012.

[26] Gilmour AR, Gogel BJ, Cullis BR, Thompson R. ASReml user guide release 3.0. VSN International Ltd, Hemel Hempstead, HP1 1ES, UK; 2009.

[27] McBratney AB, Webster R. Choosing functions for semi-variograms of soil properties and fitting them to sampling estimates. J. Soil Sci. 1986; 37: 617–639.

[28] Manly BFJ. Randomization, bootstrap and Monte Carlo methods in biology. Chapman and Hall, New York, NY; 1991.

[29] Ward JH. Hierarchical grouping to optimize an objective function. J. Amer. Statist. Assoc. 1963; 58: 236–244.

[30] Rebolledo MC, Luquet D, Courtois B, Henry A, Soulié JC, Rouan L, et al Can early vigour occur in combination with drought tolerance and efficient water use in rice genotypes? Funct. Plant Biol. 2013; 40: 582–594. 10.1071/FP12312 32481132

[31] Durel CE, Laurens F, Fouillet A, Lespinasse Y. 1998. Utilization of pedigree information to estimate genetic parameters from large unbalanced data sets in apple. Theor. Appl. Genet. 1998; 91: 1077–1085.

[32] Liebhard R, Kellerhals M, Pfammatter W, Jertmini M, Gessler C. Mapping quantitative physiological traits in apple (Malus x domestica Borkh.). Plant Mol. Biol. 2003; 52: 1497–1508. 10.1023/a:1024886500979 12956523

[33] Segura V, Denancé C, Durel CE, Costes E. Wide range QTL analysis for complex architectural traits in a 1-year-old apple progeny. Genome. 2007; 50: 159–171. 17546081 10.1139/g07-002

[34] Ben Sadok I, Moutier N, Garcia G, Dosba F, Grati-Kamoun N, Rebai A, et al Genetic determinism of the vegetative and reproductive traits in an F1 olive tree progeny. Tree Genet. Genomes 2013; 9: 205–221.

[35] Marguerit E, Bouffier L, Chancerel E, Costa P, Lagane F, Guehl JM, et al The genetics of water-use efficiency and its relation to growth in maritime pine. J. Exp. Bot. 2014; 65: 4757–4768. 10.1093/jxb/eru226 24987014 PMC4144764

[36] Avery DJ. Maximum photosynthetic rate—a case study in apple. New Phytol. 1977; 78: 55–63.

[37] Tsukaya H. Organ shape and size: a lesson from studies of leaf morphogenesis. Curr. Op. Plant Biol. 2003; 6: 57–62. 10.1016/s1369526602000055 12495752

[38] Evelan AL, Jackson DP. Sugars, signalling, and plant development. J. Exp. Bot. 2012; 63: 3367–3377. 10.1093/jxb/err379 22140246

[39] Poorter H, Niklas KJ, Reich PB, Oleksyn J, Poot P, Mommer L. Biomass allocation to leaves, stems and roots: meta-analyses of interspecific variation and environmental control. New Phytol. 2012; 193: 30–50. 10.1111/j.1469-8137.2011.03952.x 22085245

[40] Ben Sadok I, Martinez S, Moutier N, Garcia G, Leon L, Belaj A, et al Plasticity in vegetative growth over contrasted growing sites of an F1 olive tree progeny during its juvenile phase. PLoS ONE 2015; 10: e0127539 10.1371/journal.pone.0127539 26062090 PMC4465673

