# Supplementary figures and images for: Genetic Variation of Morphological Traits and Transpiration in an Apple Core Collection under Well-Watered Conditions: Towards the Identification of Morphotypes with High Water Use Efficiency
Source: PLoS One. 2015 Dec 30;10(12):e0145540. doi: 10.1371/journal.pone.0145540 (PMC4699897; doi:10.1371/journal.pone.0145540)

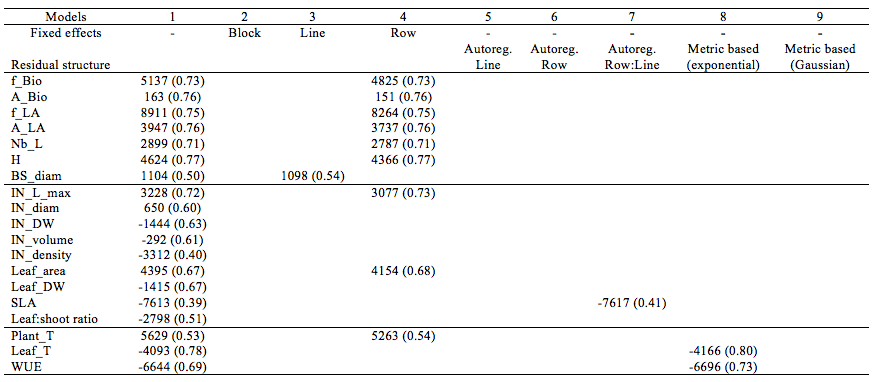

Supplement: S1 Table — When values are only presented for model 1 that means that model 1 had the lowest BIC. In all the models, the genetic effect was considered as a random effect. Refer to Table 1 for traits abbreviation. (PNG) [file pone.0145540.s001.png]

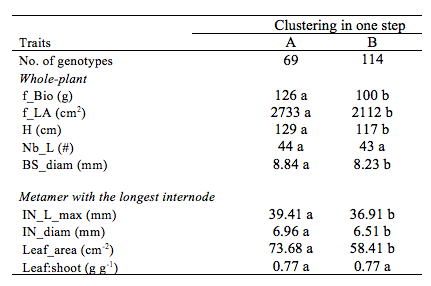

Supplement: S2 Table — For each group and trait, a one-way ANOVA was performed to estimate the cluster effects. For each variable, mean values with different letters indicates significant differences between groups according to Tukey’s test and a P<0.05. Refer to Table 1 for traits abbreviation. (PNG) [file pone.0145540.s002.png]

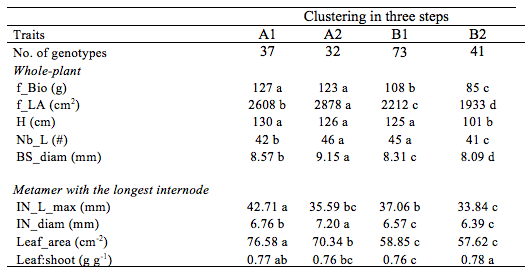

Supplement: S3 Table — For each group and trait, a one-way ANOVA was performed to estimate the cluster effects. For each variable, mean values with different letters indicates significant differences between groups according to Tukey’s test and a P<0.05. Refer to Table 1 for traits abbreviation. (PNG) [file pone.0145540.s003.png]

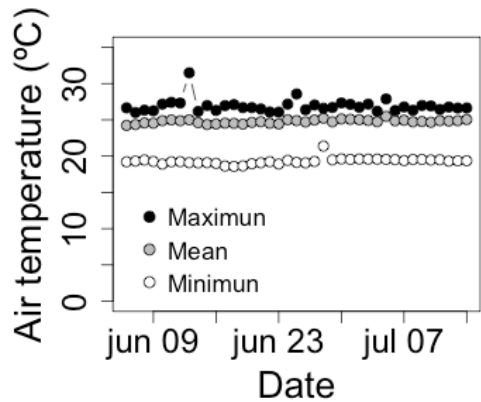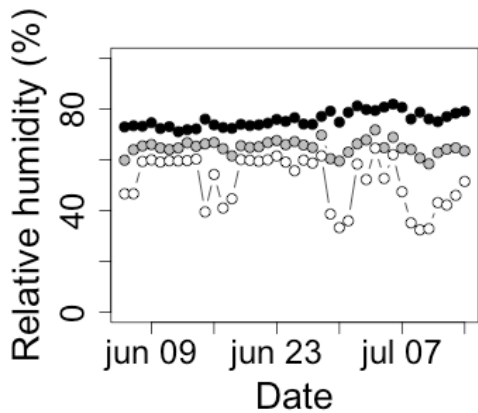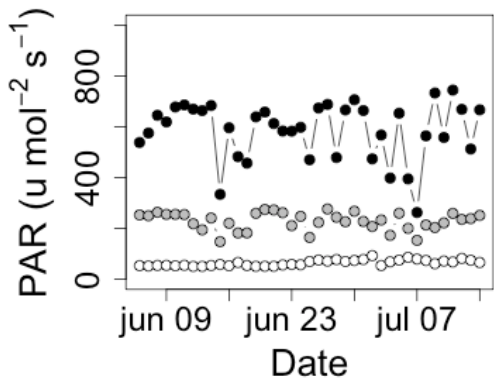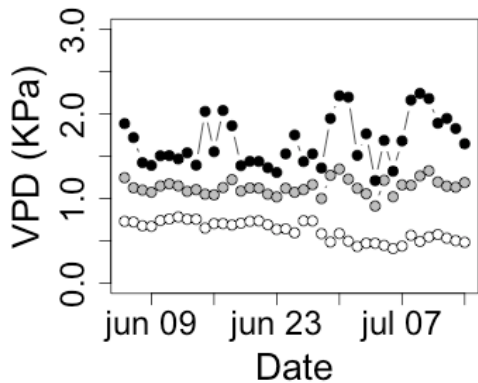

Supplement: S1 Fig — (PDF) [file pone.0145540.s004.pdf]

A

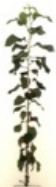

B

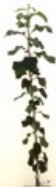

C

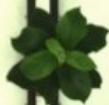

Supplement: S2 Fig — (PDF) [file pone.0145540.s005.pdf]

A

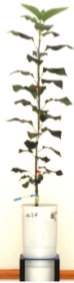

B

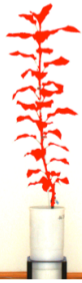

C

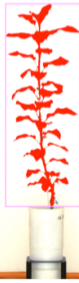

D

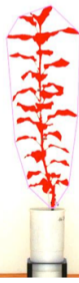

Supplement: S3 Fig — A: original plant image; B: ‘Object Sum Area’ (number of all pixels that have been identified as part of the plant); C: Object Extend (number of pixels of the width and height of the bounding box that surrounds the plant); and D: ‘Convex Hull Circumference’ (number of pixels of the area of the smallest convex envelope that contains all pixels that have been identified as part of the plant). (PDF) [file pone.0145540.s006.pdf]
