# Supplementary material for: Genetic Variation of Morphological Traits and Transpiration in an Apple Core Collection under Well-Watered Conditions: Towards the Identification of Morphotypes with High Water Use Efficiency
Source: PLoS One. 2015 Dec 30;10(12):e0145540. doi: 10.1371/journal.pone.0145540 (PMC4699897; doi:10.1371/journal.pone.0145540)
Supplement: S4 Fig — For each cluster, an example of a representative plant is presented. (PDF) [file pone.0145540.s007.pdf]

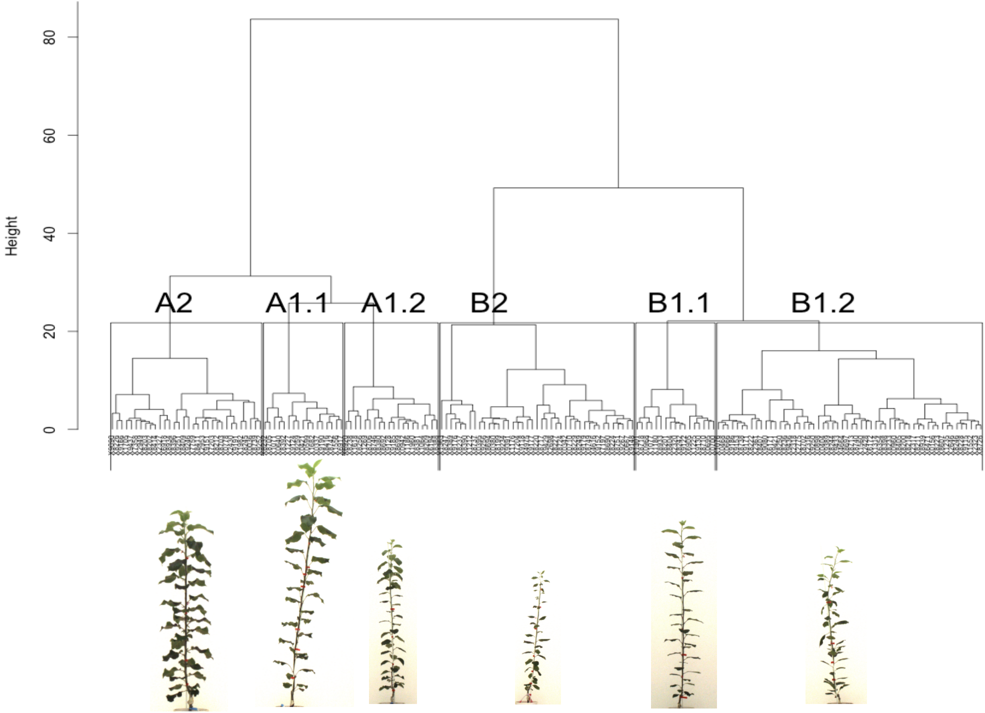
Height
80
60
40
20
0
A2
A1.1
A1.2
B2
B1.1
B1.2
